# Understanding Changes in Tomato Cell Walls in Roots and Fruits: The Contribution of Arbuscular Mycorrhizal Colonization

**DOI:** 10.3390/ijms20020415

**Published:** 2019-01-18

**Authors:** Matteo Chialva, Jonatan U. Fangel, Mara Novero, Inès Zouari, Alessandra Salvioli di Fossalunga, William G. T. Willats, Paola Bonfante, Raffaella Balestrini

**Affiliations:** 1 Department of Life Sciences and Systems Biology, University of Torino, Viale P.A. Mattioli 25, I-10125 Torino, Italy; matteo.chialva@unito.it (M.C.); mara.novero@unito.it (M.N.); ines.zouari28@gmail.com (I.Z.); alessandra.salvioli@unito.it (A.S.d.F.); paola.bonfante@unito.it (P.B.); 2 Department of Plant and Environmental Sciences, University of Copenhagen, Thorvaldsensvej 40, 1871 Frederiksberg C, Denmark; jouf@novozymes.com (J.U.F.); William.Willats@newcastle.ac.uk (W.G.T.W.); 3 School of Natural and Environmental Sciences, Newcastle University, Newcastle Upon Tyne NE1 7RU, UK; 4 Italian National Research Council (CNR), Institute for Sustainable Plant Protection (IPSP), Viale P.A. Mattioli 25, I-10125 Torino, Italy

**Keywords:** arbuscular mycorrhizal fungi, tomato, root, fruit ripening, glycan array, variance partitioning analysis

## Abstract

Modifications in cell wall composition, which can be accompanied by changes in its structure, were already reported during plant interactions with other organisms, such as the mycorrhizal fungi. Arbuscular mycorrhizal (AM) fungi are among the most widespread soil organisms that colonize the roots of land plants, where they facilitate mineral nutrient uptake from the soil in exchange for plant-assimilated carbon. In AM symbiosis, the host plasma membrane invaginates and proliferates around all the developing intracellular fungal structures, and cell wall material is laid down between this membrane and the fungal cell surface. In addition, to improve host nutrition and tolerance/resistance to environmental stresses, AM symbiosis was shown to modulate fruit features. In this study, Comprehensive Microarray Polymer Profiling (CoMMP) technique was used to verify the impact of the AM symbiosis on the tomato cell wall composition both at local (root) and systemic level (fruit). Multivariate data analyses were performed on the obtained datasets looking for the effects of fertilization, inoculation with AM fungi, and the fruit ripening stage. Results allowed for the discernment of cell wall component modifications that were correlated with mycorrhizal colonization, showing a different tomato response to AM colonization and high fertilization, both at the root and the systemic level.

## 1. Introduction

Plant cell walls are highly complex structures with sophisticated composition; they consist primarily of carbohydrates and phenolic compounds, with minor amounts of structural proteins [1]. Cell walls are dynamic structures, rather than rigid boxes, which can be remodeled during plant development, and in response to abiotic or biotic stresses [2]. Polymer structures of plant cell walls can be subjected to changes by the secretion of new polymers, combined with a large set of cell-wall-modifying proteins. The activities of these proteins can be regulated by interactions with other organisms, environmental stresses, hormones, or developmental signals [1]. Changes in the cell wall composition alter plant organ biophysical properties, thereby contributing to plant defense responses [3] and the acclimation to environmental conditions [4], with implications for crop production.

Comprehensive Microarray Polymer Profiling (CoMPP) combines the high-throughput capacity of the microarray with the specificity of monoclonal antibodies (mAbs) and carbohydrate-binding modules (CBMs), to characterize plant cell walls glycomic profiles [5,6]. Through this approach, it is possible to study the relative abundance of, and interactions between, hundreds or thousands of molecules simultaneously, using very small volumes of plant extracts. Since the first application by Møller and colleagues [7] in *Arabidopsis thaliana*, which represents a model for studying plant cell walls [8], this technique has been widely used to characterize cell wall components (mainly polysaccharides) in different plant species/tissues, performing comparative analyses across different tissues, genotypes, and growing conditions, as well as during plant interactions [9,10,11,12]. Modifications in cell wall composition, which can be accompanied by changes in its structure, were already reported during the interactions with other organisms, such as mycorrhizal fungi [13].

Arbuscular mycorrhizal fungi (AMF) are among the most widespread soil organisms that colonize the roots of land plants, where they facilitate mineral nutrient uptake from the soil in exchange for plant-assimilated carbon [14]. In arbuscular mycorrhizal (AM) symbiosis, the intracellular fungus is always surrounded by a plant-derived membrane, leading to an interfacial zone consisting of a fungal plasma membrane, a specialized interfacial matrix, and a plant membrane, called the periarbuscular membrane [13,15]. Detailed electron microscope observations have already shown that the interfacial compartment contains cell-wall like materials [13,16]. However, changes in the peripheral cell wall have also been suggested, leading to fungal accommodation inside the cell [17,18].

In addition to the improvement of host nutrition, and tolerance/resistance to environmental stresses, AM symbiosis was shown to modulate fruit features [19,20,21]. To understand the systemic effect of the fungal symbiosis on tomato fruit, an RNAseq experiment was performed on fruits collected from mycorrhizal and non-colonized fertilized plants [19]. In detail, transcriptome analysis identified several differentially expressed genes in fruits from mycorrhizal and fertilized plants; a noteworthy result was that nearly all cell wall-related genes were down-regulated in fruits from AM-colonized plants. Interestingly, an AMF-induced plant susceptibility to several antagonists that specialize in different rice tissues has been proposed [22]. Due to its role as first barrier to biotic stress factors (i.e., pathogens and pests), cell wall changes might be also related to a modification in the host susceptibility.

The CoMMP technique has been already used to verify host cell wall changes during ectomycorrhizal symbiosis [9]. Here, we applied the same approach to study the impact of AM symbiosis on the tomato cell wall composition both at the local (root) and systemic levels (fruit). Multivariate data analyses were performed on the obtained datasets looking for the effects of fertilization, AMF inoculation, and the fruit ripening stage.

## 2. Results and Discussion

Here, CoMPP was used for the first time to discern the cell-wall changes correlated with mycorrhizal colonization in tomato in both roots and fruit tissue. The ability to survey a wide range of tissues/organs already allowed for the demonstration that some glycans had highly restricted locations for specific organs, as demonstrated in *Arabidopsis* for xylogalacturonan (XGA) in siliques, flowers, and roots [7]. Here, a first experiment was setup under controlled conditions for tomato root analyses, while fruits were obtained from a previous greenhouse experiment detailed in [19]. In both experiments, plants inoculated with the AM fungus *Funnelliformis mosseae* (MYC), non-mycorrhized controls (NM), and fertilized plants (FERT) were considered. NM and MYC plants received the same fertilization treatment (Long–Ashton nutrient solution) at a modified phosphorous level, to favor AM symbiosis establishment. The FERT condition was adopted as a full nutrient reference condition and the commercial fertilizer “Asso di Fiori” (CIFO s.r.l.) was applied. Results obtained on roots collected under “NM”, “MYC” and “FERT” conditions, and on berries from “MYC” and “FERT” conditions at four different ripening stages (mature green, breaker, turning, and red) were analyzed, plus berries from NM plants at the red ripe stage (R). Indeed, in this experiment, under NM conditions, probably due to the low nutrient regime applied to favor mycorrhization, plants were not able to produce a large amount of fruits as reported in Zouari et al. [19]. In the first experiments, before cell wall alcohol insoluble residue (AIR) preparation, the colonization rate was evaluated in the first experiment, showing that MYC plants were well-colonized, with a mean frequency value (F) of 78.2% (Supplementary Figure S1). Mycorrhization parameters in the second trial were already measured in Zouari et al. [19] and the F values were of 65%.

### 2.1. Mapping of the Plant Cell Wall Polymers in Tomato Roots and Fruits

**Figure 1:**
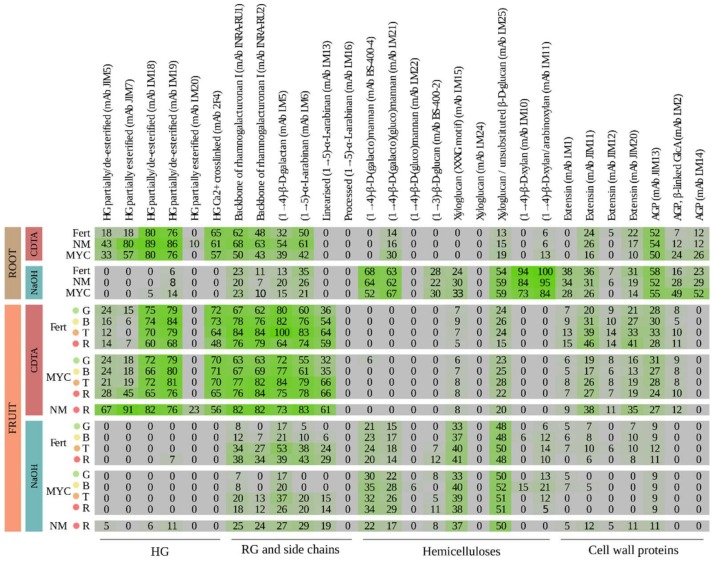
CoMPP (comprehensive microarray polymer profiling) analysis of CDTA- (Cyclohexane Diamine Tetraacetic Acid) and NaOH-extracted cell-wall fractions from tomato root and fruit tissues under mycorrhizal (MYC), fertilized (FERT) and non-mycorrhizal (NM) conditions. The heatmap shows the relative abundance of the epitopes. Fruits were analyzed at four different ripening stages: “mature green” (G), “breaker” (B), “turning” (T), and “red” (R). The highest signal in the data set was set to 100, and all of the other values were normalized accordingly. A cut-off of 5 was introduced.

Using Cyclohexane Diamine Tetraacetic Acid (CDTA) and NaOH as extractants, cell-wall polysaccharides were sequentially removed from the roots of MYC and NM plants, which had received the same nutritional solution, as well as from roots of non-mycorrhized plants grown under high fertilization (FERT). The CoMPP profiles are reported as a heatmap in Figure 1, pointing to a typical cell wall pattern for angiosperms [7,23]. General models of the primary plant cell wall in dicotyledonous and some monocotyledonous plants typically report that the cellulose microfibrils are cross-linked with hemicelluloses, including mannans, xylans, mixed-linkage glucans (MLG), and xyloglucans. This network is then further embedded in a matrix of pectic polysaccharides, including homogalacturonan (HG) and rhamnogalacturonan-I (RG-I), joint, with a small amount of glycoproteins [23,24]. In more detail, in dicot plants, the primary cell-wall consists of 30% each of cellulose, matrix glycans and pectins, plus 1–10% of structural proteins. In particular, in dicots, pectins can account up to 60% of cell wall mass in fleshy fruits [25]. Recently, Cornault et al. [26,27] reported the cell wall profiles in different Solanaceous species, including tomato. Here, combining the data across the two extractions, an overview of the changes was obtained in the cell-wall epitope levels in MYC roots, in comparison with non-colonized tomato roots from plants maintained at two different fertilization levels (NM, FERT). The present data are in agreement with the results obtained through detailed previous electron microscope observations of the root interfacial compartment [15]. In situ techniques (enzymes, lectins, and antibodies) have allowed for the localization of cell wall-like material in this compartment, and to localize β-1,4-glucans, non-esterified homogalacturonans, xyloglucans, proteins rich in hydroxyproline (HRGPs), arabinogalactan proteins (AGPs), and expansins at the interface in several different plant/AM fungus combinations, as well as on peripheral cell walls [15,17,28,29,30,31,32]. As a further step, we performed microscopy observations of tomato MYC roots.

**Figure 2:**
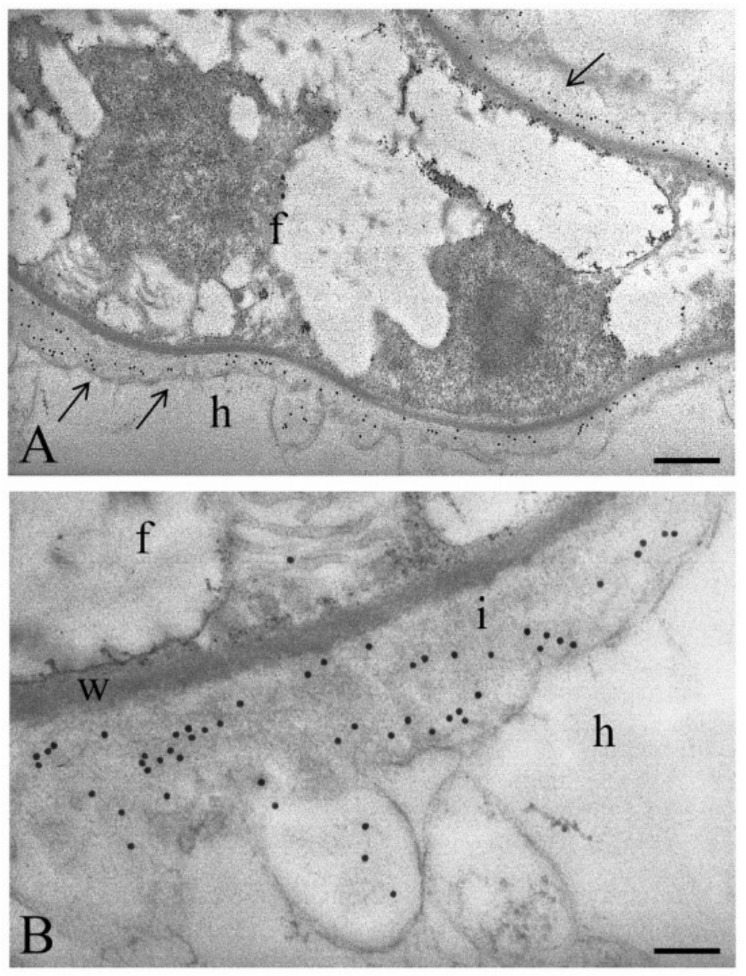
JIM5 labelling to localize homogalacturonans on ultra-thin sections of tomato root colonized by an arbuscular mycorrhizal fungus. (**A**) At the electron microscope level, a new apoplastic compartment (i.e., the symbiotic interface), based on host membrane proliferation, is evident around the intracellular hyphae. Gold granules (arrows) are present in the interface space around the intracellular fungus (f). Bar, 0.55 μm; (**B**) Magnification of the interface region (i), labelled after treatment with JIM5. f, intracellular hypha; h, host cell; w, fungal cell wall. Bar, 0.15 μm.

Until now, few data suggest that morphological changes occur in AM-colonized roots of tomato [33,34]. Our results support previous findings on other host plant/AM fungus combinations [13], showing the interface compartment around the intracellular hyphae (Figure 2A), and the presence of molecules typical of plant cell walls, such as de-esterified homogalacturonans recognized by the JIM5 antibody (Figure 2B).

To understand whether the local impact of the mycorrhizal fungus on plant cell wall metabolism also manifested at a systemic level, we obtained CoMPP profiles from tomato fruits. Results showed changes at the cell-wall epitope levels in fruits collected from MYC, in comparison with fruits from non-colonized tomato plants maintained at two different fertilization levels (NM, FERT). Even if several papers showed the impact of the AM symbiosis on tomato fruit quality [35,36] and on metabolic reprogramming occurring in several plant tissues [37,38], no data were provided to date on the impact of the AM fungus on tomato cell wall composition.

Variance partitioning analysis (VPA), as implemented in the vegan::varpart function, on the whole root glycome showed that *F. mosseae* inoculation explained 19% and 14% of the total variance (*p* < 0.001) for CDTA and NaOH fractions, respectively (Supplementary Figure S2). Similarly, fertilization level also significantly explained 20% and 16% of the variance (*p* < 0.001) for the CDTA and NaOH datasets, respectively. The total contribution of each variable for each factor is summarized in Figure S2C–D.

### 2.2. The Root Glycome Is Shaped by Both Nutrient Levels and AM Symbiosis

**Figure 3:**
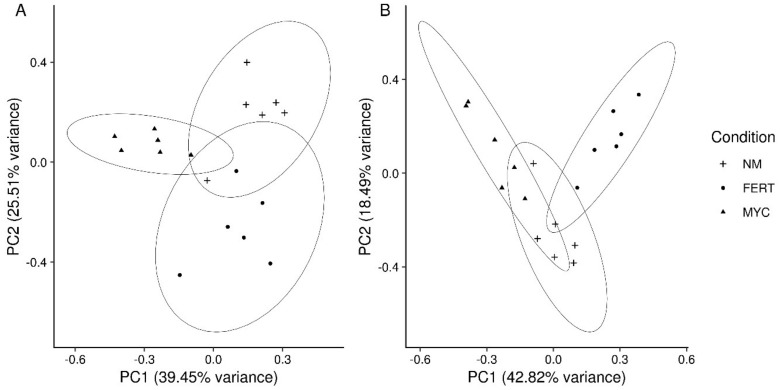
Principal Component Analysis (PCA) of the tomato roots glycome under mycorrhizal (MYC), fertilized (FERT), and non-mycorrhizal (NM) conditions. (**A**) Analysis of the CDTA-extracted fraction; (**B**) Analysis of the NaOH-extracted fraction. The first two components clustered by condition were plotted, and 95% confidence ellipses were drawn (*n* = 6).

Considering roots, a first explorative principal component analysis (PCA) was performed in order to detect the variation between the conditions. Plots showed that biological replicates clustered according to the treatment in both the CDTA and NaOH datasets with a small overlap between groups (Figure 3).

**Figure 4:**
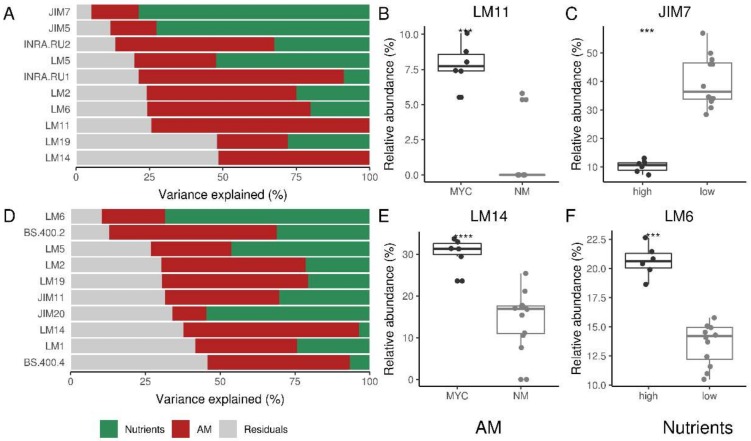
Variance partitioning analysis (VPA) of tomato roots glycome under mycorrhizal (MYC), fertilized (FERT) and non-mycorrhizal (NM) conditions. (**A**,**D**) Variance explained by each factor (AM colonization and fertilization treatment) for the top 10 more correlated antibodies for CDTA and NaOH fraction, respectively. The most correlated antibody for each factor is depicted in boxplots for the CDTA (**B**,**C**) and NaOH (**E**,**F**) fractions. Significant differences according to Kruskal–Wallis tests were reported with asterisks (**** *p* ≤ 0.0001, *** *p* ≤0.001).

Using VPA, as implemented in the variancePartition R package [39], we were further able to detect the explained variance by each of the considered factor (AM occurrence and fertilization level) for each variable (antibodies). The analysis allowed us to pick out epitopes whose abundance well correlates with AMF inoculation and/or nutrients level (Figure 4).

It is worth noting that the variability of the epitopes related to pectins was largely explained by high fertilization, and secondarily, by AM symbiosis in both the CDTA and NaOH datasets. Indeed, within the CDTA extraction, all of the homogalacturonan (HG) epitopes yielded the highest signals for the NM condition, and again, the rhamnogalacturonans I (RG-I)-related epitopes are higher in NM samples, and lower in MYC compared to FERT, except for LM5, where the signal values were very similar between MYC and FERT (Figure 1). Epitopes recognized by JIM7 (partially methyl-esterified HG) and LM6 (1,5-α-L-arabinan) were those in which the fertilization level explained the major amount of variance for CDTA and NaOH data, respectively (Figure 4A,D). However, the first one was more abundant in low nutrient treatments while the second correlated with higher nutrient treatment (Figure 4C,F). Interestingly, some other HG/RG mAbs displayed a similar pattern, such as JIM5 and LM5, being much more decreased in the FERT condition than in the MYC condition (Figure 1). As shown in Figure 2C, epitopes recognized by JIM5 were detected in the interface region around the intracellular fungus, in part explaining the higher signal in the MYC roots with respect to the FERT ones. However, some mAbs recognizing HG (LM18, LM19) and RG (INRA.RU2) showed similar intensities in the FERT and MYC conditions, and for most of them, both factors (nutrients and AMF presence) showed similar contributions in explaining their variance (Figure 4).

Conversely, *F. mosseae* inoculation (in MYC treatment) explained a large part of hemicellulose (mannans, xyloglucans, and xylan) variability, at least in CDTA data. The highest percentage of the explained variance was found in LM11 (1,4-β-d-xylan/arabinoxylan), which resulted in a high over-representation in MYC roots (Figure 4B). However, as well as for cell-wall proteins, NaOH extraction showed a better resolution. Xylan (LM10, LM11) yielded the highest signal score in the dataset, increasing in the FERT samples and decreasing in the MYC compared to NM. These data are in agreement with previous observations showing the regulation of genes involved in hemicellulose remodelling in mycorrhizal roots (reviewed in [13]).

Finally, structural proteins such as HRGPs and AGPs were mainly affected in MYC treatment. Interestingly, despite a similar contribution to the variance of cell-wall proteins, the role of mycorrhization was different for extensin proteins and AGPs (Figure 4). All extensins were lower in the MYC condition when compared to FERT and NM, in both the CDTA and NaOH datasets (Figure 1). By contrast, AGPs were more abundant in the MYC samples with the exception of the epitope recognized by JIM13. LM14 mAbs explained the highest amount of variance (Figure 4) and LM2 showed a similar trend, with a decrease in the FERT sample in both datasets.

CoMPP results well-supported previous observations showing a different cell-wall component accumulation in MYC roots, i.e., at the interface mirrored by the up-regulation of genes involved in plant cell wall synthesis [13]. It has also been reported that some transcripts were specifically localized in arbusculated cells [18,40,41], suggesting both a role in the interface creation, and in cell expansion during arbuscule development [15]. According to this hypothesis, the molecular mechanisms activated by the fungal presence and leading to the construction of the interface compartment might also have an additional target, namely the peripheral cell wall [18]. Although the approach that we followed efficiently detected significant changes in the cell wall composition of MYC roots compared to NM, it should be considered that MYC samples are a mixture of colonized and non-colonized root regions. In ectomycorrhizal roots, Sillo and colleagues [9] suggested that the observed reduction in all the cell wall polysaccharide groups could also result from a dilution of plant material, due to the presence of cell wall material of fungal origin in colonized roots. Although it is not possible to identify a reference epitope that can be used to normalize data, and considering that several epitopes did not vary considerably among the treatments, an important dilution of the plant wall material in the AM-colonized root samples does not seem to exist.

Although the antibodies that were used were monoclonal, and the specificities for plant cell wall components had already been published [7], the higher signal for β-1,3-glucan in ectomycorrhizal samples has been correlated to its presence in the fungal cell wall [9]. In our experiment, the values for this component are similar in the three treatments. It is worth noting that AM fungal cell wall becomes progressively thinner during the intracellular phase, reaching a thin amorphous structure in the thinner arbuscular branches. The presence of β-1,3-glucan has not been always observed in the thin cell wall of the arbuscules, depending on the fungal species [13]. Since information on its presence in *F. mosseae* cell wall is not available, an absence of this component in the *F. mosseae* cell wall, at least at the symbiotic stage, can be hypothesized, although we cannot exclude that the fungal cell wall fraction at this stage is not a very important impact on the results.

In contrast to previous studies, the present set up also included a “fully fertilized” thesis (FERT), which provided the plants with a higher level of nutrients compared to the NM control. Interestingly, MYC and FERT patterns differed significantly, suggesting a different impact on the plant cell wall components in the presence of the AM fungus. The complex picture highlighted here by CoMPP on roots from both MYC and FERT plants suggests that the changes observed in the presence of the fungus are not exclusively due to the improvement in the fertilization state. Although we have no data on the nutrient levels in the roots from the two treatments (MYC vs. FERT), it is worth noting that fruits from MYC and FERT plants have been previously reported to have a similar content in phosphorous (P), potassium (K), and sulfur (S), while the MYC ones had a slight decrease in carbon (C) and nitrogen (N) contents [19]

### 2.3. AM Symbiosis, Nutrient Levels, and Ripening Stages Modulate the Fruit Glycome

**Figure 5:**
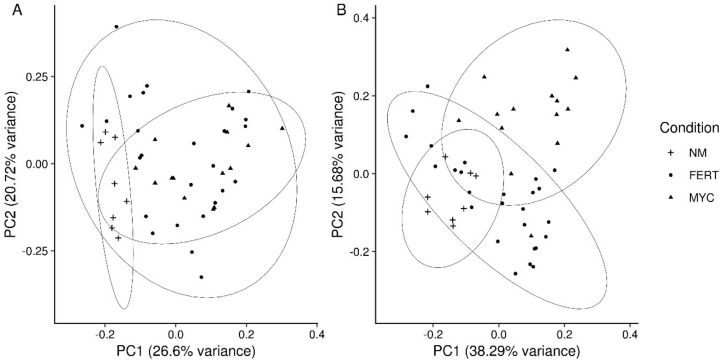
Principal Component Analysis (PCA) of the tomato fruits glycome under mycorrhizal (MYC), fertilized (FERT), and non-mycorrhizal (NM) conditions. (**A**) Analysis of the CDTA-extracted fraction; (**B**) Analysis of the NaOH-extracted fraction. The first two components, clustered by condition, were plotted, and 95% confidence ellipses were drawn (*n* = 6).

The analysis of the fruit glycome under AM colonization revealed a complex pattern: all the tested factors (fungal colonization, ripening stage, and nutrients) seemed to play a relevant role. From a preliminary PCA ordination, we detected a slightly different pattern between the extraction type and a clear clustering according to conditions was only evident in NaOH dataset (Figure 5).

As for the root dataset we applied VPA, confirming that all of the three factors successfully explained a significant amount of global variance with a relevant part of the unexplained variance. *F. mosseae* inoculation explained 9% and 8% of the total variance (*p* < 0.01) in the CDTA and NaOH fractions, respectively. The fruit ripening stage explained 11% and 32% of total variance (*p* < 0.01) respectively for CDTA and NaOH fractions, while the nutrient level significantly explained 31% of variance (*p* < 0.01) only in CDTA dataset (Supplementary Figure S3). The total contribution of each variable for each factor is summarized in Figure S3C–D.

The Knowledge of the changes in cell wall composition in fruits is essential for understanding the role of enzyme-driven action during fruit development and softening [42]. Here, the aim was to evaluate the impact of AM symbiosis on the cell wall polysaccharides, also considering different ripening stages. An impact of the AM fungi on the transcriptome profile and the amino acid composition of the tomato fruit has, in fact, already been reported [19,20]. Additionally, the molecular basis of fruit ripening has been extensively studied in tomato [43,44,45,46].

Here, we have showed that in fruit tissue, the main polysaccharides detected in the CDTA extraction were homogalacturonans (HG) (JIM5, JIM7, LM18, LM19, LM20 and 2F4), rhamnogalacturonans-I (RG-I) (INRA-RU1, INRA-RU2, LM5, LM6 and LM13) and glycoproteins (JIM11, JIM12, JIM20, JIM13 and LM2), in line with previous results obtained on tomato fruits [47]. In particular, HGs and RGs-I showed strong signals, and revealed the highest variation across experimental conditions. In the NaOH fraction, no HG epitopes were detected, and a weak signal for RG-I and glycoproteins also emerged (as already extracted in the first fraction), while an increased signal for hemicelluloses as mannan epitopes (BS-400-4, LM21, LM22) and xyloglucan emerged.

**Figure 6:**
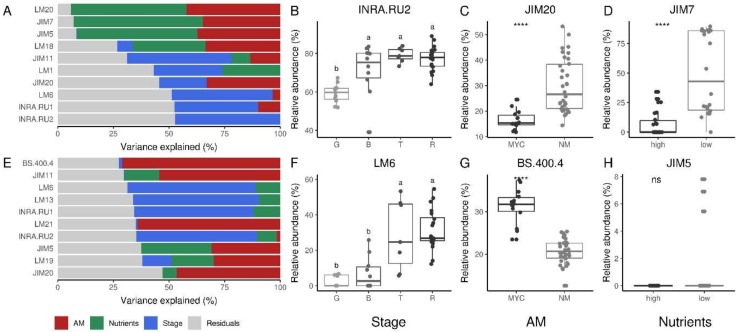
Variance partitioning analysis (VPA) of the tomato fruits glycome under mycorrhizal (MYC), fertilized (FERT), and non-mycorrhizal (NM) conditions. (**A**,**E**) Variance explained by each factor (ripening stage, AM colonization, and fertilization treatment) for the top 10 best correlated antibodies for the CDTA and NaOH fraction, respectively. The most well-correlated antibody for each factor is depicted in boxplots for CDTA (**B**–**D**) and NaOH (**F**–**H**) fractions. Fruits were analyzed at four different ripening stages: “mature green” (G), “breaker” (B), “turning” (T), and “red” (R). Significant differences according to Kruskall–Wallis tests were reported with asterisks (**** *p* ≤ 0.0001) or letters (data indicated with different letters are statistically different, ns = not-significant differences).

In both datasets, the variance explained by the ripening stage was higher in the RG epitopes (INRA.RU1, INRA.RU2, and LM6), with a lower contribution by the other two factors (Figure 6). INRA.RU2 and LM6, respectively, for the CDTA and NaOH datasets, were in fact the mAbs where the major amount of variance is explained by the ripening stage factor (Figure 6B,F). Using both mAbs, the relative intensity increased towards the fruit ripening process from the green ripe to the red ripe stage, with a statistically supported decrease in their abundances in the early ripening stages (mature green) (Figure 6B,F).

Interestingly, when considering the AM status, the higher amount of related variance in the CDTA-extracted fraction was explained by HG epitopes (JIM5, JIM7, LM18, LM20) but not RGs. Further, a slight increase in their abundance across the ripening process emerged in MYC, but not in the FERT condition (Figure 1), suggesting again an impact of the AM fungus independently from the nutritional level. As previously cited in fact, the nutritional levels were similar in the fruits from the two treatments [19]. The NaOH phase held a deeper resolution for hemicelluloses in fruit tissue. Particularly, we found that AM inoculation almost exclusively explained the variances of LM21 and BS.400.4 mAbs. Indeed, BS.400.4 was the Ab in which AM inoculation explained the major amount of variance in NaOH fraction (see Figure 6G). Interestingly, the abundances of both (1-4)-β-d mannans (LM21 and BS.400.4) was higher in the MYC samples when compared to the FERT ones, and it did not seem to correlate with the ripening stage factor. By contrast, no differences were detected in xylan and xyloglucan in both datasets.

Similarly, the nutrients factor also impacted common categories such as HGs. In particular, JIM7 was the mAb with a higher fraction of variance being explained by the nutrient level (Figure 6D) in the CDTA fraction. At last, we compared the CoMPP data with gene RNA-seq expression profiles obtained in a previous study by Zouari et al. [19] on the same materials that we analyzed where fruit from the MYC plant were compared with that of FERT plants at the red ripe stage. Here, among DEGs (differentially-expressed genes) a number of cell-wall related transcripts were found, as reported in Table S1. Particularly, we denoted a massive down-regulation of pectinesterase-coding genes in MYC fruits with respect to the FERT condition.

In agreement with these data, JIM5 signal (de-esterified HGs) were similar in MYC and FERT conditions, except at the red ripe stage, while JIM7 mAb (partially esterified HGs) revealed a higher abundance in MYC condition at the breaker (B) and the Red ripe stage (Supplementary Figure S4). By contrast, considering relative abundances of both these mAbs in MYC samples (G, B, and T ripening stages), JIM5 held higher values compared to JIM7, being in contrast with what we described. However, the obtained profiles of the polymer changes showed that most of the variations took place between mid- and full ripening, showing a consistent difference only at the red ripe stage (R).

A general suppression of cell wall metabolism in the grape berry skin during ripening has been also observed, with genes involved in pectin metabolism that showed much more heterogeneous transcriptional behavior [48]. In the context of our experiment (Table S1), it has to be taken into account that our analyses have been performed on the whole pericarp and the activation of genes expressed only in specific berry tissues could be masked due to a dilution effect or a post-transcriptional activity of pectinesterase activity. On the other hand, the up-regulation of several transcripts coding for enzymes that are involved in gluconeogenesis matched with the higher abundance of hemicelluloses mAbs (BS.400.4, LM21) [19]. The regulation of genes involved in cellulose, hemicellulose, and pectin metabolism has again been reported during the maturation in grape berries [48].

To correlate the gene expression data and the cell-wall changes with fruit features, morphometrical measurements on tomato berries from three conditions (MYC, NM and FERT) were analyzed (Supplementary Figure S5). Fruits from MYC plants showed lower pericarp thickness (PT) and mean diameter (MD) compared with fruits from NM plants. No significant differences were noted for these parameters between fruits of NM and FERT plants. Additionally, under MYC conditions, fruit fresh weight (FFW) was also lower, compared to the FERT condition but under the NM condition. Seed number (SN) was significantly lower in both MYC and NM conditions, while the pericarp weight (PW) and the circularity index (CI) were not influenced by experimental conditions.

At our knowledge, tomato fruit morphometrical parameters in mycorrhizal vs non-mycorrhizal plants have been poorly investigated even if other fruit parameters, such as total yield, fruit mass, or seeds number and weight were often measured [49,50]. In our previous work [19], MYC plants showed a longer fruiting period, resulting in a higher fruit production compared with both NM and FERT plants. Our current data showed that some of the measured parameters do not respond to AM inoculation (PW, CI), and others were highly responsive (FFW, PT, MD), while the seed number (SN) only responded to the fertilization level, as expected. Interestingly, all of the parameters that were specifically modulated by mycorrhizal status, fruit diameter, weight, and pericarp thickness had a strong correlation with fruit growth, rather than fruit ripening [51,52] suggesting that fruit development and not only ripening is probably deeply impacted by mycorrhization. Additionally, fruit shape and size traits (such as FFW, PT and MD) have been linked to the turgor of parenchyma cells, which is closely related to cell wall features [25,53]. However, further morphological detailed analyses are needed to correlate the observed changes with cell wall modification.

Interestingly, concerning glycoproteins, only a few regulations have emerged. VPA analysis indeed detected a role for the ripening stage, such as in JIM11 and JIM20, recognizing extensins. Moreover, the same mAbs held a fraction of the variance explained by AMF inoculation (Figure 6C).

Until now the complex it is known that hemicellulose and pectin networks are modulated during fruit ripening, but the mechanisms involved are still largely unknown [25]. Although it is known that cell wall properties are an important determinant of fruit texture [27], further analyses could be performed to verify whether the different cell wall modification induced by MYC and FERT treatments might be correlated with a difference in morphometric traits and textural properties. Moreover, the detected changes in fruit cell wall upon AM inoculation could also have a role in fruit postharvest shelf life.

## 3. Materials and Methods

### 3.1. Plant Material and Sampling

Tomato plant (*Solanum lycopersicum* L.) materials used for CoMPP analysis were obtained in two different pot experiments. Root material was obtained from plants grown under controlled conditions in the growth chamber, while fruits were collected from greenhouse grown plants. In both assays, *cv*. Moneymaker was used, since it responds well to arbuscular mycorrhizal symbiosis (AM), as previously reported [19,54]. Tomato seeds were germinated under axenic condition according to Salvioli et al. [20]. Briefly, seeds were surface-sterilized in 70% ethanol (with the addition of 3–4 drops of Tween 20) (3 min) following a step in 5% commercial hypochloride in sterile dH_2_O (13 min) and three washes in sterile dH_2_O water (10 min each). Seeds were then transferred in petri dishes with 0.6% Plant agar (Duchefa, Haarlem, The Netherlands) medium, and germinated in the dark at 23 °C for five days. Seedlings were then moved to day/night conditions for another four days (16 h light (23 °C)/8 h dark (21 °C)) and then transferred to pots.

To obtain root material, mycorrhizal plants (MYC) were inoculated with a *Funneliformis mosseae* (BEG12, formerly *Glomus mosseae*) commercial inoculum, containing AM fungal propagules (spores, mycelium and mycorrhizal root pieces) in a carrier of mixed inert mineral, purchased from MycAgro Lab. (Dijon, France). The granular inoculum was 30% diluted in sterile quartz sand and pumice mixture. Control non-mycorrhizal (NM) and fertilized (FERT) plants received the same substrate without the AM inoculum.

Under growth chamber conditions, plants were grown in medium-sized pots (10 × 10 × 12 cm) under controlled conditions (14 h light (24 °C)/10 h dark (20 °C)) for 90 days, until full AM colonization was achieved. Pots were watered once a week with a modified Long–Ashton nutrient solution containing a middle-strength phosphorus concentration (30 μM Na_2_HPO_4_), and once a week with tap water for the MYC and NM condition. By contrast, FERT condition plants were watered once a week with a commercial fertilizer solution “Asso di Fiori” (CIFO s.r.l., S. Giorgio di Piano, Italy) containing 3.8 mM P, 12.9 mM N, 6.6 mM S, and 3.8 mM K, and once a week with tap water. After 90 days, roots were sampled and divided into two portions the first was used to assess the mycorrhizal colonization (see below), and the second was immediately frozen at −80 °C for glycomic analyses.

To obtain the fruits, a greenhouse experiment was conducted in large-sized plastic pots (14 × 14 × 16 cm) under the same conditions reported in Zouari et al. [19]. MYC and NM plants were watered with a modified Long-Ashton solution at (300 μM Na_2_HPO_4_), while the FERT plants were watered with the commercial fertilizer solution “Asso di Fiori”.

Due to different timings and plant phenological stages in the two experiments (vegetative versus reproductive), different phosphorous levels were adopted. In the first experiment, which lasted only three months, P-fertilization was kept as low as possible (32 μM). In the second trial, since we aimed to reach the fruit-set stage with a growing season longer than six months, which requires a higher amount of nutrients, NM and MYC plants were kept at 300 μM phosphorous as a good balance between tomato growth/fruiting and mycorrhiza establishment. However, NM plants had a very poor yield, and they produced a very limited number of fruits.

Fruits were harvested, from MYC and FERT plants, at four different ripening stages: mature Green (35 days after pollination (dap), indicated with G), Breaker (40 dap, indicated with B), Turning (42 dap, indicated with T) and Red ripe (55 dap, indicated with R). Due to the poor yield in the NM plants, only fruits at the R stage were collected from these plants. After collection, the fruits were cut in half with a sterile scalpel, seeds, and placental tissue were discarded, and the resulting pericarp was stored at −80 °C until processing.

At the end of each experiment, mycorrhizal colonization was assessed on three plants for each condition, according to Trouvelot et al. [55]. Roots were washed in tap water to remove sand particles, and a representative portion of each plant was stained for 12 h in 0.1% (*w*/*v*) cotton blue in lactic acid, and de-stained in pure lactic acid for at least 4 h. For each plant, 60 cm were observed. No evidence of AM colonization was found in the NM and FERT condition in both experiments as cross-contamination.

### 3.2. Cell-Wall Preparation

Collected tomato fruit and root tissues were used to prepare alcohol-insoluble residues (AIR) for CoMPP analysis. Tissues were placed in 2-mL tubes, freeze-dried overnight, and homogenized into powder using TissueLyser (QIAGEN, Hilden, Germany). Samples were then washed in 70% ethanol under rotation at room temperature (RT) three times for 30 min, and two times for 60 min. After each wash supernatant was removed by centrifugation (13,000 rpm, 10 min.). A final wash for 10 min in 100% acetone was performed as described above, and the resulting AIR pellet was dried at RT.

### 3.3. Comprehensive Microarray Polymer Profiling

CoMPP was carried out as described in Møller et al. [7]. Cell wall polymers were extracted sequentially from 10 mg of AIR with 300 µL of 50 mM CDTA, pH 7.5, and 4 M NaOH with 0.1% (*v*/*v*) NaBH_4_, and spotted on a nitrocellulose membrane with a pore size of 0.45 μm (Whatman, Maidstone, UK) using an Arrayjet Sprint (Arrayjey, Roslin, UK). Each sample was printed with two technical replicates and four dilutions, and probed as described in Pedersen et al. [56]. The complete list of monoclonal antibodies used in this study is reported in Table 1. The arrays were scanned using a flatbed scanner (CanoScan 8800 F, Canon, Søborg, Denmark) at 2400 dpi, and quantified using Array-Pro Analyzer 6.3 (Media Cybernetics, Rockville, MD, USA). The data is presented as a heatmap, where each value is an average of the two replicates and four dilutions for each sample. The highest value is set to 100, and all other values normalized accordingly. Additionally a cut-off of 5 was introduced.

### 3.4. Fruit Morphometry and Biometry

Fruit morphometric measurements were performed using the Tomato Analyzer v.3 software [57,58,59]. Images of equatorial fruit sections were acquired using an Epson Perfection 2450 photo flatbed scanner at 600 dpi, and further processed with the software. For each condition, a minimum of 15 fruits were analyzed, averaging measured parameters from the two slices of each fruit. For each experimental condition, the pericarp thickness (PT), the mean diameter (MD), and circularity index (CI) were analyzed. Additionally, the fruit fresh weight (FFW), the pericarp weight (PW) and the seed number (SN) were measured.

### 3.5. Morphological Observations

Tomato roots were prepared for light (LM) and transmission electron microscopy (TEM), according to Balestrini et al. [31]. Briefly, root segments 0.5 cm long were fixed in 2.5% (*v*/*v*) glutaraldehyde in phosphate buffer (0.5 mM pH 7.4), post-fixed in 1% (*w*/*v*) osmium tetroxide in the same buffer, dehydrated in an ethanol series of 30, 50, 70, 90, 100% (*v*/*v*) (15 min each step) at room temperature and then embedded in LR White resin (Polysciences, Warrington, PA, USA). Semi-thin sections (1 μm) were stained with 1% (*w*/*v*) toluidine blue for morphological observations, while thin sections (about 70 nm) were treated with an antibody against partially de-esterified homogalacturonans (JIM5), according to Sillo et al. [9], and counterstained with uranyl acetate and lead citrate. Sections were observed using a CM 10 Transmission Electron Microscope (Philips, Eindhoven, The Netherlands).

### 3.6. Multivariate Statistical Analyses

Multivariate analysis was applied in order to evaluate the influence of AMF colonization and nutrient levels on the tomato root glycome. Ripening stage was also considered as a factor in the fruit dataset. Variance partitioning analysis (VPA) was applied to calculate the contribution of each factor to global glycomic profiles using the “varpart” function in “vegan” package [60]. The contribution of individual fractions was tested on the Redundancy analysis (RDA) model using permutational ANOVA (*p* < 0.05, 999 permutations). The contribution of single epitopes to the variance of each factor was tested using the “variancePartition” package [39], following instructions reported on the package’s vignettes. Both VPA analyses were performed on normalized intensity values, as suggested in Hoffman and Schadt [39].

All analyses were performed by using custom scripts in R [61] and data visualized using ggplot2 library [62].

## 4. Conclusions

In this study, we report the comprehensive measurement of cell wall polymer composition in tomato roots and fruits, providing an atlas that represents a baseline for evaluating the impact of the AM symbiosis on tomato cell wall composition, both at the local (root) and systemic (fruit) levels.

Taking advantage of multivariate statistics and particularly of VPA, we were able to finely dissect tomato root and fruit glycomes under mycorrhizal colonization, describing in detail the influence of each factor on the CoMPP data set. Our study demonstrates that such methods, even if they were specifically designed for gene expression studies, are also sound and reliable in other contexts, allowing researchers to understand complex data sets and their biological meanings. Additionally, our data elegantly showed the different tomato responses to AM colonization and a high fertilization treatment, both at the root and systemic levels, in term of cell wall component modifications. However, further studies will be needed to verify whether the different impact of these treatments on the fruit cell wall components might influence its quality features, considering the relationship between fruit texture (and softening) and cell wall metabolism.

## Figures and Tables

**Table 1:** List of monoclonal antibodies (mAbs) used in this study. HG: homogalacturonan.

Target	mAb	Description
Pectins	JIM5	partially/de-esterified HG
	JIM7	partially esterified HG
	LM18	partially/de-esterified HG
	LM19	partially/de-esterified HG
	LM20	partially esterified HG
	2F4	Ca^2+^ crosslinked HG
	INRA-RU1	Backbone of rhamnogalacturonan I
	INRA-RU2	Backbone of rhamnogalacturonan I
	LM5	(1→4)-β-d-galactan
	LM6	(1→5)-α-l-arabinan
	LM13	Linearized (1→5)-α-l-arabinan
	LM16	Processed (1→5)-α-l-arabinan
Hemicelluloses	BS-400.4	(1→4)-β-d-(galacto) mannan
	LM21	(1→4)-β-d-(galacto)(gluco) mannan
	LM22	(1→4)-β-d-(gluco) mannan
	BS-400.2	(1→3)-β-d-glucan (callose)
	LM15	Xyloglucan (XXXG motif)
	LM24	Xyloglucan (mAb LM24)
	LM25	Xyloglucan/unsubstituted β-d-glucan
	LM10	(1→4)-β-d-xylan
	LM11	(1→4)-β-d-xylan/arabinoxylan
Glycoproteins	LM1	Extensin
	JIM11	Extensin
	JIM12	Extensin
	JIM20	Extensin
	JIM13	Arabinogalactan protein (AGP)
	LM2	Arabinogalactan protein (AGP) β-linked GlcA
	LM14	Arabinogalactan protein (AGP)

## Supplementary Materials

Supplementary Materials can be found at http://www.mdpi.com/1422-0067/20/2/415/s1. Figure S1: Mycorrhizal colonization in *S. lycopersicum* cv. Moneymaker roots at 90 days after inoculation under climate-controlled chamber conditions. Figure S2: Variance partitioning analysis (VPA) of the tomato root glycome under mycorrhizal (MYC), fertilized (FERT) and non-mycorrhizal (NM) conditions. Figure S3: Variance partitioning analysis (VPA) of the tomato fruits glycome under mycorrhizal (MYC), fertilized (FERT) and non-mycorrhizal (NM) conditions. Figure S4: Relative abundances of JIM5 (A) and JIM7 (B) mAbs in mycorrhizal (Myc) and fertilized (FERT) tomato fruits at the four ripening stages tested, “mature green” (G), “breaker” (B), “turning” (T), and “red” (R). Figure S5: Fruit morphometric parameters measured on fresh fruits or extracted from berry equatorial sections with TomatoAnalyzer software. Table S1. Differentially expressed genes involved in cell-wall-related processes in Mycorrhized (MYC) vs Fertilized (FERT) tomato fruits (Zouari et al., 2014).

## Abbreviations

AGPs	Arabinogalactan proteins
AM	Arbuscular mycorrhiza
AMF	Arbuscular mycorrhizal fungi
CDTA	Diamino-cyclo-hexane-tetra-acetic acid
CoMPP	Comprehensive microarray polymer profiling
dap	Days after pollination
HGs	Homo-galacturonans
HRGPs	Hydroxyproline-rich glycoproteins
mAB	monoclonal antibody
PCA	Principal component analysis
RGs	Rhamno-galacturonans
VPA	Variance partitioning analysis
