# Supplementary material for: Understanding Changes in Tomato Cell Walls in Roots and Fruits: The Contribution of Arbuscular Mycorrhizal Colonization
Source: Int J Mol Sci. 2019 Jan 18;20(2):415. doi: 10.3390/ijms20020415 (PMC6359600; doi:10.3390/ijms20020415)
Supplement: Supplementary file 1 [file ijms-20-00415-s001.pdf]

Supplementary Figures

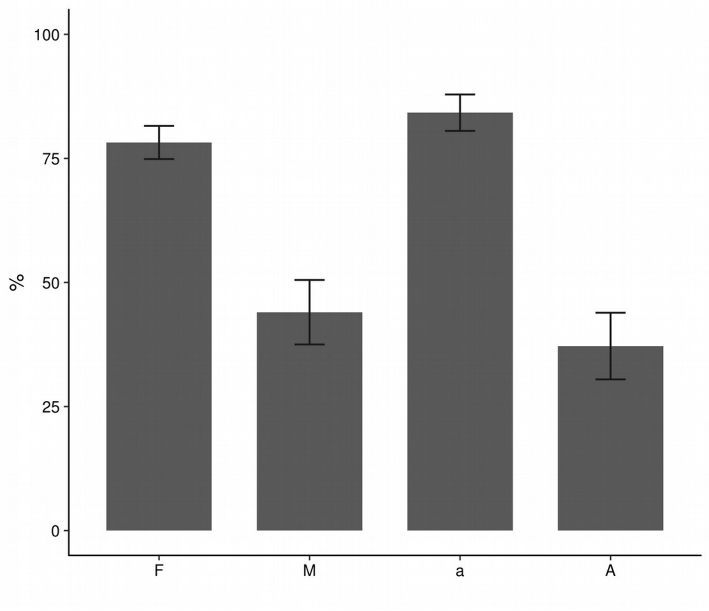

Figure S1. Mycorrhizal colonization in *S. lycopersicum* cv. Moneymaker roots at 90 days after inoculation under climate-controlled chamber conditions. F, frequency of mycorrhization; M, intensity of mycorrhization in the root apparatus; a, presence of arbuscules within the colonized portions; A, presence of arbuscules in the root apparatus. Values are in percentage and standard deviation is drawn (n=3).

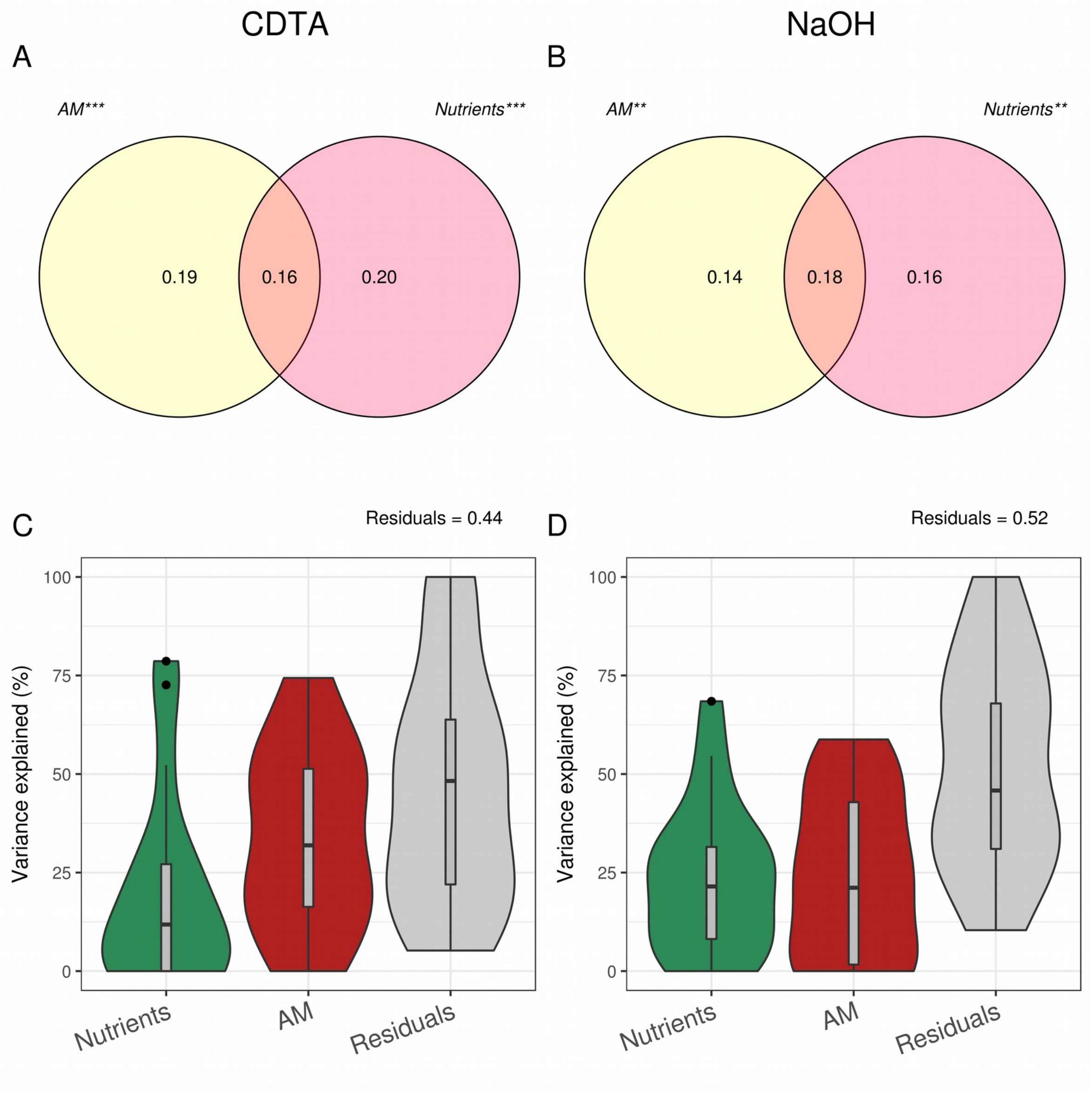

Figure S2. Variance partitioning anaysis (VPA) of tomato root glycome under mycorrhizal (MYC), fertilized (FERT) and non-mycorrhizal (NM) conditions. (**A**,**B**) Partitioning of the global variance by factor and their collinearity as obtained from vegan::varpart R function for CDTA and NaOH cell wall fractions respectively. Numbers in venns represents the portion of explained variance by each factor alone. Statistical significance of single factors was tested on RDA model using permutational ANOVA (999 permutations; * P<0.05; ** P<0.005; *** P< 0.001). (**C**,**D**) Violin plots representing the distribution of variance explained by epitopes by factor for CDTA and NaOH cell wall fractions respectively.

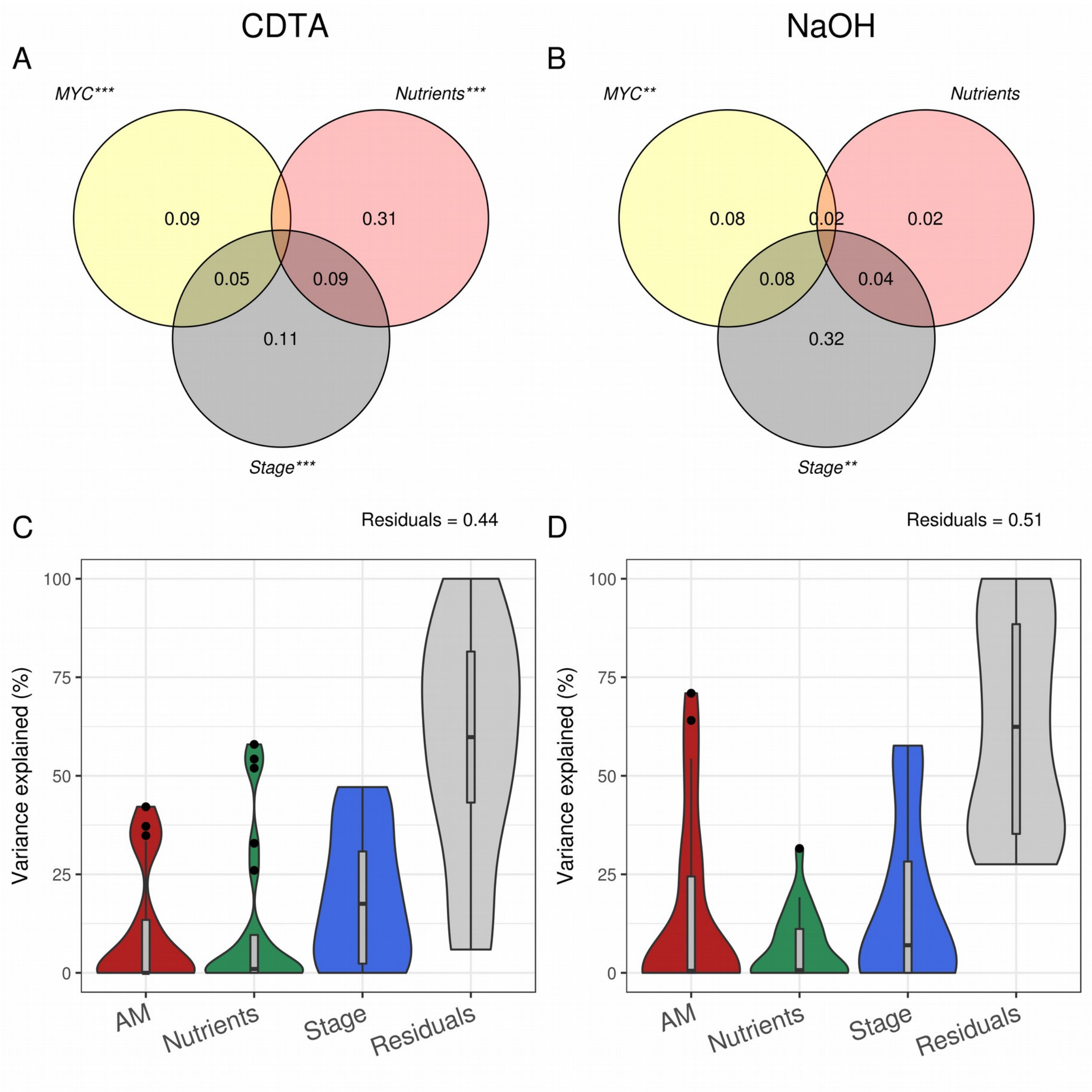

Figure S3. Variance partitioning anaysis (VPA) of tomato fuits glycome under mycorrhizal (MYC), fertilized (FERT) and non-mycorrhizal (NM) conditions. (A,B) Partitioning of the global variance by factor and their collinearity as obtained from vegan::varpart R function for CDTA and NaOH cell wall fractions respectively. Numbers in venns represents the portion of explained variance by each factor alone. Statistical significance of single factors was tested on RDA model using permutational ANOVA (999 permutations; * P<0.05; ** P<0.005; *** P< 0.001). (C,D) Violin plots representing the distribution of variance explained by epitopes by factor for CDTA and NaOH cell wall fractions respectively.

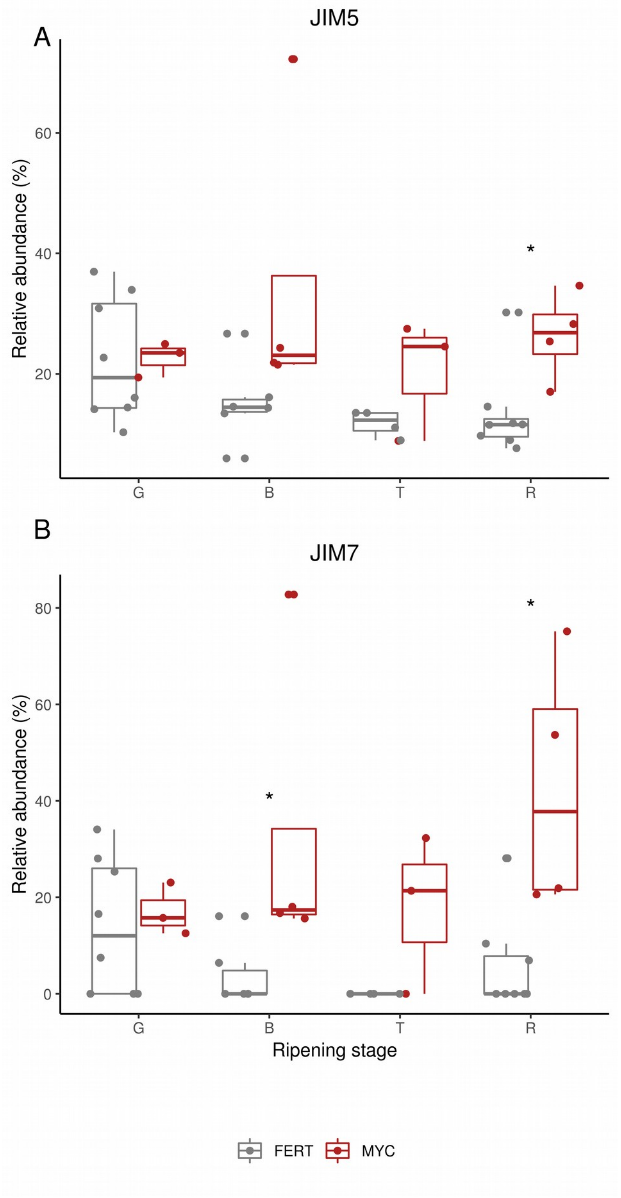

Figure S4. Relative abundances of JIM5 (A) and JIM7 (B) mAbs in mycorrhizal (Myc) and Fertilized (FERT) tomato fruits in the four ripening stages tested, 'mature green' (G), 'breaker' (B), 'turning' (T) and 'red' (R). Significant differences according to Kruskal-Wallis test were reported with asterisks (P ≤ 0.05).

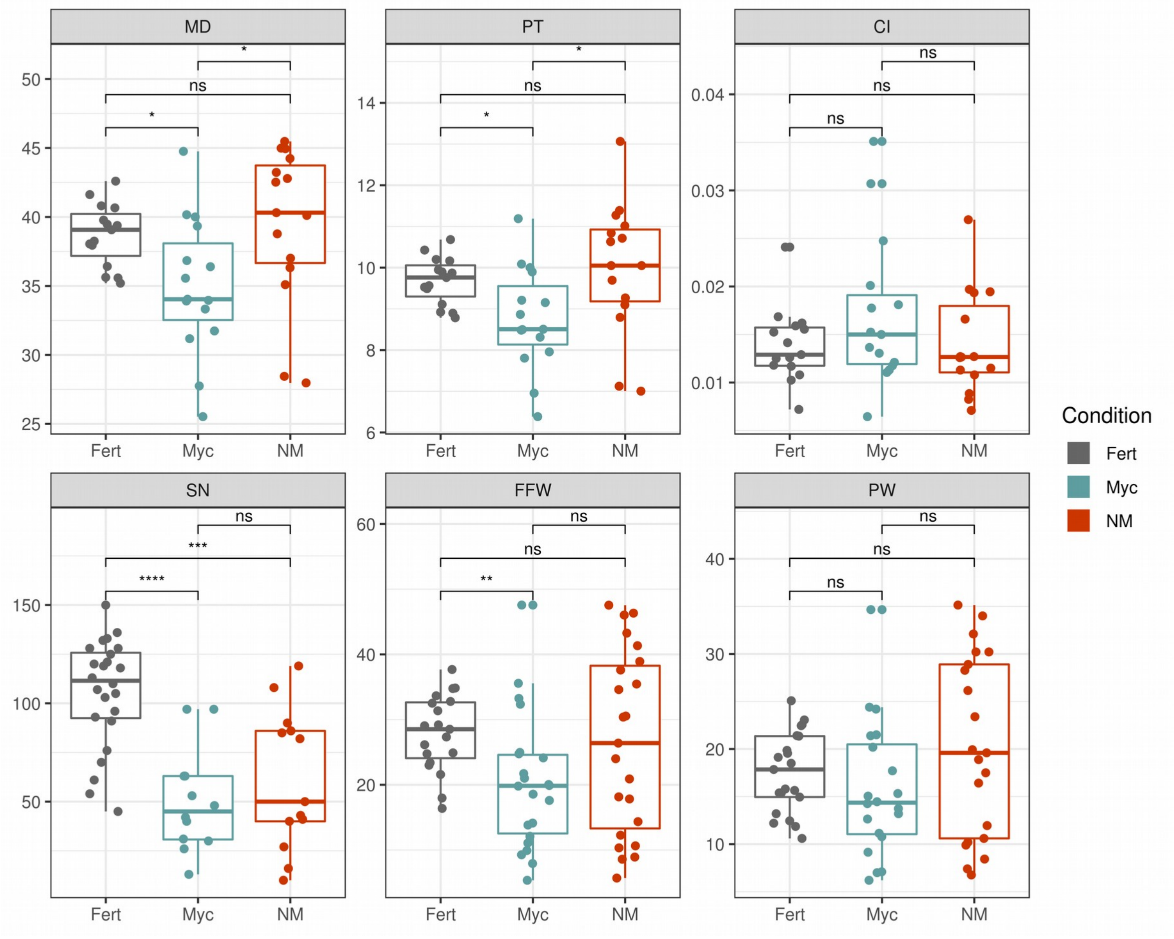

Figure S5. Fruit morphometric parameters measured on fresh fruits or extracted from berry equatorial sections with TomatoAnalyzer software. Mycorrhizal (MYC), fertilized (FERT) and non-mycorrhizal (NM) conditions were considered. Mean diameter (MD, mm), pericarp thnickness (PT, mm), circularity index (CI), seed number (SN), fresh weight (FW, g) and pericarp weight (PW, g) were measured. Statistical testing was applied considering fertilized plants (FERT) as reference group and performing pairwise t-tests (*, $P \leq 0.05$; **, $P \leq 0.01$; ***, $P \leq 0.001$; ****, $P \leq 0.0001$).

Supplementary Tables

Table S1. Differentially expressed genes involved in cell-wall related processes in Mycorrhized (MYC) vs Fertilized (FERT) tomato fruits (Zouari et al., 2014).

Transcript ID	log_2fold-change	adjusted p-value	Annotation
Solyc10g083290.1	4,36	0,006	Cell-wall invertase
Solyc02g062340.2	3,24	0,000	Fructose-bisphosphate aldolase, class-I
Solyc09g011810.2	3,16	0,000	Fructose-1 6-bisphosphatase class 1
Solyc02g091560.2	2,61	0,000	Serine hydroxymethyltransferase
Solyc12g015770.1	2,19	0,002	Cellulose synthase
Solyc01g110360.2	1,84	0,000	Fructose-bisphosphate aldolase, class-I
Solyc03g058950.2	1,56	0,046	Inositol hexakisphosphate and diphosphoinositol-pentakisphosphate kinase 2
Solyc07g045160.2	1,54	0,001	Pyrophosphate-dependent phosphofructokinase TP0108
Solyc04g071340.2	1,47	0,003	Fructose-1 6-bisphosphatase class 1
Solyc10g086730.1	1,36	0,002	Fructose-1 6-bisphosphatase class 1
Solyc08g082440.2	1,18	0,010	UDP-glucose 4-epimerase
Solyc02g084440.2	1,17	0,001	Fructose-bisphosphate aldolase
Solyc03g118410.2	1,15	0,039	Acyl carrier protein (ACP)
Solyc05g052600.2	1,09	0,006	Chloroplast sedoheptulose-1,7-bisphosphatase
Solyc07g048090.1	1,01	0,035	Cell adhesion protein (Fasciclin-like arabinogalactan protein 2)
Solyc06g051930.2	0,89	0,088	Pyruvate kinase family protein
Solyc07g062530.2	-0,83	0,089	Phosphoenolpyruvate carboxylase 2
Solyc03g113030.2	-0,83	0,082	Aldose 1-epimerase-like protein
Solyc06g083310.2	-0,87	0,073	Glycosyl transferase, family 8
Solyc02g092790.2	-1,03	0,010	Putative arabinogalactan-protein
Solyc12g056580.1	-1,08	0,033	Cellulose synthase
Solyc02g030230.2	-1,12	0,057	UDP-glucose 4-epimerase
Solyc07g064170.2	-1,15	0,002	Pectinesterase 1
Solyc07g041750.2	-1,28	0,002	Protein of unknown function DUF246
Solyc04g073990.2	-1,28	0,000	Annexin
Solyc04g071650.2	-1,29	0,023	Cellulose synthase
Solyc02g088630.2	-1,30	0,018	Glycosyl transferase, family 8
Solyc09g091430.2	-1,33	0,001	Pectate lyase 1-27
Solyc03g123630.2	-1,35	0,042	Pectinesterase/pectinesterase inhibitor U1
Solyc02g084720.2	-1,41	0,002	Beta-galactosidase

Solyc02g084720.2	-1,41	0,002	Beta-galactosidase
Solyc03g115500.2	-1,57	0,001	Heparanase
Solyc06g051800.2	-1,62	0,000	Expansin 1 protein
Solyc03g083910.2	-1,64	0,000	Acid beta-fructofuranosidase
Solyc08g005800.2	-1,66	0,000	Pectinacetylesterase like protein
Solyc08g005800.2	-1,66	0,000	Pectinacetylesterase like protein
Solyc07g056000.2	-1,71	0,001	Xyloglucan endotransglucosylase/hydrolase 7
Solyc02g072150.2	-1,74	0,000	Alpha alpha-trehalose-phosphate synthase (UDP-forming)
Solyc03g097050.2	-1,76	0,000	Cellulose synthase-like protein
Solyc07g053640.1	-2,01	0,000	Arabinogalactan-protein
Solyc02g090360.2	-2,10	0,000	Laccase-22
Solyc11g007690.1	-2,19	0,000	Pyruvate kinase
Solyc03g093130.2	-2,28	0,000	Xyloglucan endotransglucosylase-hydrolase XTH3
Solyc01g099630.2	-2,37	0,000	Probable xyloglucan endotransglucosylase/hydrolase 1
Solyc01g105070.2	-2,44	0,001	Cationic peroxidase
Solyc03g093120.2	-2,49	0,000	Xyloglucan endotransglucosylase/hydrolase 9
Solyc01g080280.2	-2,54	0,000	Glutamine synthetase
Solyc03g093080.2	-2,67	0,000	Xyloglucan endotransglucosylase/hydrolase 9
Solyc06g009190.2	-4,25	0,004	Pectinesterase
Solyc03g123620.2	-5,22	0,000	Pectinesterase
